# Synthesis of Amphiphilic Copolymers of *N*-Vinyl-2-pyrrolidone and Allyl Glycidyl Ether for Co-Delivery of Doxorubicin and Paclitaxel

**DOI:** 10.3390/polym14091727

**Published:** 2022-04-23

**Authors:** Anna Nechaeva, Alexander Artyukhov, Anna Luss, Mikhail Shtilman, Inessa Gritskova, Anton Shulgin, Mikhail Motyakin, Irina Levina, Efrem Krivoborodov, Ilya Toropygin, Evgeniy Chistyakov, Leonid Gurevich, Yaroslav Mezhuev

**Affiliations:** 1 Department of Biomaterials, Mendeleev University of Chemical Technology of Russia, Miusskaya Sq., 9, 125047 Moscow, Russia; anechaeva16@gmail.com (A.N.); artyukhow@yandex.ru (A.A.); al.luss@yandex.ru (A.L.); shtilmanm@yandex.ru (M.S.); vv1992@yandex.ru (E.K.); ewgenijj@rambler.ru (E.C.); 2 Department of Chemistry and Technology of Macromolecular Compounds, MIREA—Russian Technological University (RTU MIREA), 119454 Moscow, Russia; inessagritskova@gmail.com (I.G.); antonshulgin2017@yandex.ru (A.S.); 3 Emanuel Institute of Biochemical Physics, Russian Academy of Sciences, 119334 Moscow, Russia; motyakin@hotmail.com (M.M.); iilevina@inbox.ru (I.L.); 4 Semenov Federal Research Center for Chemical Physics, Russian Academy of Sciences, 119991 Moscow, Russia; 5 V.N. Orekhovich Institute of Biomedical Chemistry, Russian Academy of Sciences, 119121 Moscow, Russia; toropygin@rambler.ru; 6 Department of Materials and Production, Aalborg University, Skjernvej 4A, 9220 Aalborg, Denmark; lg@mp.aau.dk

**Keywords:** amphiphilic copolymers, drug delivery, paclitaxel, doxorubicin, cancer therapy

## Abstract

Co-delivery of chemotherapeutics in cancer treatment has been proven essential for overcoming multidrug resistance and improving the outcome of therapy. We report the synthesis of amphiphilic copolymers of *N*-vinyl-2-pyrrolidone and allyl glycidyl ether of various compositions and demonstrate that they can form nanoaggregates capable of simultaneous covalent immobilization of doxorubicin by the epoxy groups in the shell and hydrophobic-driven incorporation of paclitaxel into the core of nanoparticles. The structure of the obtained copolymers was characterized by ^13^C NMR, IR, and MALDI spectroscopy, as well as adsorption at the water/toluene interface. A linear increase in the number-average molecular weight of amphiphilic copolymers and a decrease in the number-average diameter of macromolecular aggregates with an increase in the ratio *N*-vinyl-2-pyrrolidone/allyl glycidyl ether were observed. The assembled nanocarriers were characterized by DLS. The reported novel nanocarriers can be of interest for delivery and co-delivery of a wide range of pharmacological preparations and combined therapy for cancer and other deceases.

## 1. Introduction

In 2020, oncological diseases were detected in 19.3 million people and caused the death of almost 10 million people [1]. Although a number of drugs are known to be used used in chemotherapy, their application is complicated by low bioavailability and toxic side effects. The use of polymer carriers for anticancer drugs opens up new possibilities in cancer treatment by optimizing and vectorizing the pharmacological action, decreasing toxicity and increasing bioavailability of drugs [2,3,4,5]. The application of polymer nanoparticles, hydrogels [6], amphiphilic co-network gels [7,8,9,10] and other macromolecular systems [11,12] allows for a significant increasing in the bioavailability and a decreasing in the toxic effects of pharmacologically active drugs. One of the approaches to the preparation of drug carriers is related to the use of amphiphilic copolymers capable of self-assembly with formation of nanoparticles [13]. Block copolymers [14] and graft copolymers [15] with hydrophilic fragments based on polyethylene oxide, as well as poly(N-vinylcaprolactam), poly(*N*-vinyl-2-pyrrolidone) and hydrophobic fragments based on polyglycolide, polylactide and poly(ε-caprolactone) [16,17,18,19], are used as amphiphilic polymer carriers for the formation of nanoparticles.

Recently, the authors developed an approach to the synthesis of linear amphiphilic polymers based on low-molecular-weight poly(*N*-vinyl-2-pyrrolidone) with hydrophilic alkyl end groups. The amphiphilic low-molecular-weight poly(*N*-vinyl-2-pyrrolidone) proved to be effective in terms of loading with hydrophobic substances [20,21] and does not display any significant toxicity [22,23]. However, the amphiphilic *N*-vinyl-2-pyrrolidone homopolymer is relatively ineffective as a carrier for drugs with high solubility in water [24], which limits the possibilities of its application in combination therapy. Combination chemotherapy is one of the leading approaches in the treatment of a number of cancer types [25]. Therefore, the present article is dedicated to the synthesis and investigation of the properties of a new amphiphilic copolymer of *N*-vinyl-2-pyrrolidone containing chemically reactive allyl glycidyl ether units. The introduction of the allyl glycidyl ether residues into the copolymer carrier chain enables covalent immobilization of water-soluble drugs containing nucleophilic functional groups. Doxorubicin meets these requirements and was therefore selected as the model nucleophilic water-soluble anticancer drug.

Doxorubicin, a common broad-spectrum drugs, has shown high efficacy in the treatment of breast cancer [26], lung cancer [27], gastric cancer [28], sarcoma [29], lymphoma [30], neuroblastoma [31], glioblastoma [32] and other oncological diseases [33]. Doxorubicin both intercalates into DNA [34] and stimulates the formation of radicals that damage cell membranes [35], which determines its antitumor activity. Its high cardiotoxicity limits its application in treatment of oncological diseases [36,37,38]. Minimization of cardiotoxic effects and the implementation of combination therapy are the main goals in using nanosized carriers for doxorubicin immobilization [39]. As carriers of doxorubicin, nanosystems based on boron nitride [40], lipids [41], dextran [42], PLGA [43], polydopamine [44], iron oxide [45], gold [46], collagen and poly(3-acrylamidophenylboronic acid) [47] have been reported in the literature. The reported strategies involve the controlled release of doxorubicin, which has a cumulative dose [48] and limits the available treatment options. In this respect, the strategy based on covalent immobilization of doxorubicin through an amino group not involving the pharmacophore anthracycline fragment [33] appears to be the most promising in terms of reducing cardiac toxicity.

The hydrophobic paclitaxel, which was found to be especially effective in combination therapy with doxorubicin [49,50,51], was chosen as the second model anticancer drug. Although the use of pure paclitaxel is hampered by its negligible solubility in water, low bioavailability and development of myelosuppression and peripheral neuropathy [52], its inclusion in nanocarriers can significantly increase the therapeutic effect [53]. In the present article, the hydrophobic core of the aggregates of an amphiphilic copolymer of *N*-vinyl-2-pyrrolidone and allyl glycidyl ether containing covalent-immobilized doxorubicin were loaded with paclitaxel. The use of amphiphilic copolymers of *N*-vinyl-2-pyrrolidone and allyl glycidyl ether opens up possibilities for the immobilization of any drug combination in which one drug is sufficiently nucleophilic and the other is hydrophobic.

## 2. Materials and Methods

Monomers of *N*-vinyl-2-pyrrolidone (VP) and allyl glycidyl ether (AGE) were purchased from Sigma-Aldrich and additionally purified by vacuum distillation. 1,4-dioxane was purchased from KhimMed (Moscow, Russia), dried over sodium and distilled under a vacuum. N-octadecylmercaptan and azobisisobutyronitrile (AIBN) from Sigma-Aldrich (St. Louis, MO, USA) were used without further purification. Doxorubicin hydrochloride (Synbias Pharma, Kyiv, Ukraine) and paclitaxel (LC Laboratories, New Boston, MA, USA) were used for immobilization. Triethylamine (KhimMed, Moscow, Russia) was used as a catalyst for covalent immobilization of doxorubicin by epoxy groups of the amphiphilic copolymer. The amphiphilic copolymer of *N*-vinyl-2-pyrrolidone and allyl glycidyl ether were isolated by precipitation into diethyl ether (KhimMed, Moscow, Russia). Dialysis was performed using a 500 Da molecular weight cutoff membrane (Labware supplier store 500 MWCO, Darmstadt, Germany).

The structure of the synthesized compounds was characterized by ^13^C NMR in DMSO-d6 medium using a Bruker Avance 500 (Bruker, Zurich, Switzerland, New Materials and Technologies Research Center at IBCP RAS), IR spectroscopy in KBr pellets using a Nicolet 380 (Thermo Scientific, Waltham, MA, USA), MALDI tablets (Ultraflex II, Bruker, Karlsruhe, Germany, mass spectrometry studies were performed using the “Human Proteome” resource center equipment, Moscow, Russia) by desorption with Nd:YAG laser (355 nm) and UV-vis spectroscopy (Eppendorf BioSpectrometer, Hamburg, Germany). Particle size distributions were recorded by dynamic laser light scattering NANO-flex II (Colloid Metrix, Meerbusch, Germany). 

### 2.1. Synthesis of an Amphiphilic Copolymer of N-Vinyl-2-pyrrolidone and Allyl Glycidyl Ether

In an ampoule, 10 g (0.09 mol) of *N*-vinyl-2-pyrrolidone and a specified amount of allyl glycidyl ether were dissolved in 20 mL of 1,4-dioxane. Then, 0.1 g (6 × 10^−4^ mol) of AIBN and 0.286 g (10^−3^ mol) of n-octadecylmercaptan were dissolved in the obtained mixture of co-monomers. The ampoule was purged with argon and sealed and then kept for 20 h at 343 K. The product was isolated by precipitation in a tenfold volume excess of diethyl ether. The isolated copolymer of *N*-vinyl-2-pyrrolidone and allyl glycidyl ether was dried under vacuum at room temperature until constant weight was achieved. The number-average molecular weight was determined by reverse iodometric titration after oxidation of terminal sulfide groups with m-chloroperbenzoic acid, as described previously [54]. Copolymerization was carried out for different amounts of allyl glycidyl ether in the reaction system: 0.01 mol, 0.02 mol, 0.03 mol, and 0.05 mol.

### 2.2. Investigation of the Adsorption of the Amphiphilic Copolymer of N-Vinyl-2-pyrrolidone and Allyl Glycidyl Ether on the Water/Toluene Interface

Surface tension isotherms and critical aggregation concentrations (CAC) at 296 K were obtained from adsorption of amphiphilic copolymers of *N*-vinyl-2-pyrrolidone and allyl glycidyl ether at the water/toluene interface using a KRÜSS DSA30 (KRÜSS, Hamburg, Germany) particle shape analyzer and the pendant drop method. Adsorption was calculated using the Gibbs adsorption equation; the limiting value of Gibbs adsorption corresponding to the complete monolayer (Г_max_) was determined from the slope of the linear dependence plotted in the coordinates “C/Г vs. C”. Calculation of the effective thickness of the adsorption layer (δ) and the effective area occupied by an individual amphiphilic copolymer macromolecule on the interfacial surface (S) was carried out using Equations (1) and (2), respectively.

(1) $$\delta =\frac{{Г}_{\max}M}{\rho },$$

(2) $$S=\frac{1}{{Г}_{\max N_{A}}}$$

where Г_max_ is the maximum value of Gibbs adsorption; N_A_ is the Avogadro number; and M and ρ are the molecular weight and density of the copolymers, respectively. 

CAC was determined by the intersection method in the coordinates “σ vs. lnC”. 

### 2.3. Immobilization of Doxorubicin with an Amphiphilic Copolymer of N-Vinyl-2-pyrrolidone and Allyl Glycidyl Ether

Immobilization was carried out in aqueous medium as a result of the reaction of 0.129 g of doxorubicin hydrochloride and 1 g of an amphiphilic copolymer of *N*-vinyl-2-pyrrolidone and allyl glycidyl ether (VP/AGE = 3). Catalysis was achieved by the addition of 0.022 g of triethylamine. The reaction system was placed in a sealed ampoule, purged with argon and kept for 5 days. The doxorubicin immobilization product was purified by dialysis against water using a 500 MWCO membrane (Labware supplier store, Darmstadt, Germany).

### 2.4. Incorporation of Paclitaxel into an Amphiphilic Copolymer Containing Covalently Immobilized Doxorubicin

An amount of 0.2 g of a copolymer of *N*-vinyl-2-pyrrolidone and allyl glycyl ether with a terminal n-octadecylthio group and a molecular weight of 1850 Da was dissolved in 10 mL of distilled water. After 2 min of ultrasonic treatment, 1 mL of a solution of paclitaxel in chloroform at a concentration of 5 × 10^−3^ g·mL^−1^ was added to the solution of the amphiphilic copolymer carrier, and the resulting emulsion was subjected to ultrasonication for 18 min. Then, chloroform was removed by vacuum distillation in a rotary evaporator; excluded paclitaxel was separated by filtration through a 0.45 µm filter.

## 3. Results and Discussion

**Scheme 1:**
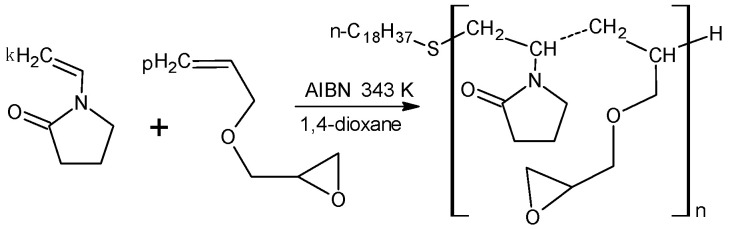
Synthesis of an amphiphilic copolymer of *N*-vinyl-2-pyrrolidone and allyl glycyl ether with a terminal *n*-octadecyl group.

The synthesis of amphiphilic copolymers of *N*-vinyl-2-pyrrolidone and allyl glycyl ether was carried out by a radical copolymerization reaction with AIBN as an initiator and in the presence of *n*-octadecylmercaptan as a chain transfer agent (Scheme 1).

**Figure 1:**
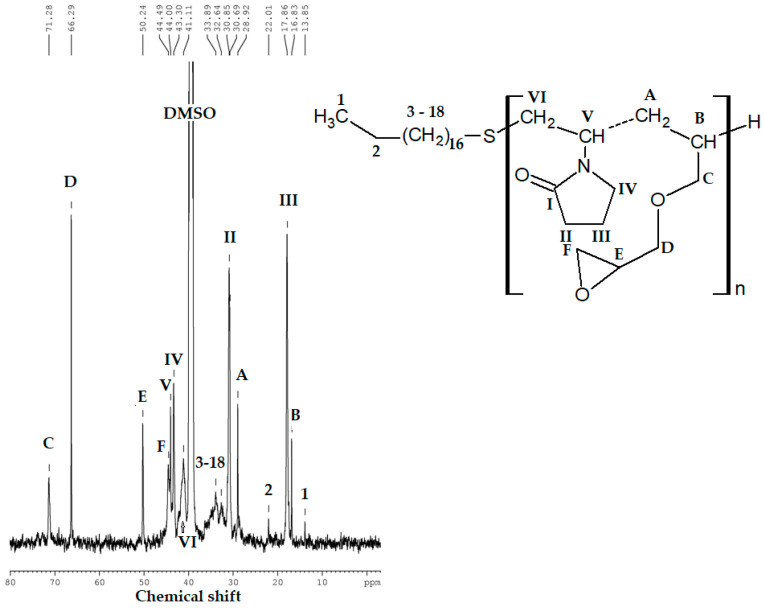
^13^C NMR spectrum of an amphiphilic copolymer of *N*-vinyl-2-pyrrolidone and allyl glycyl ether with a terminal *n*-octadecyl group recorded in DMSO-d6 medium.

The structure of the synthesized copolymer was characterized by ^13^C NMR, IR and MALDI spectroscopy. The assignment of signals in the ^13^C NMR spectrum of the synthesized amphiphilic copolymer is shown in Figure 1 (the signal of carbon atom I of the pyrrolidone ring has a chemical shift of 172.81 ppm) and is consistent with the presence of *N*-vinyl-2-pyrrolidone and allyl glycidyl ether residues in the chain, as well as the terminal *n*-octadecyl group.

When assigning the signals, we used the literature data on the analysis of ^13^C NMR spectra of polymers and copolymers of *N*-vinyl-2-pyrrolidone [55,56].

**Figure 2:**
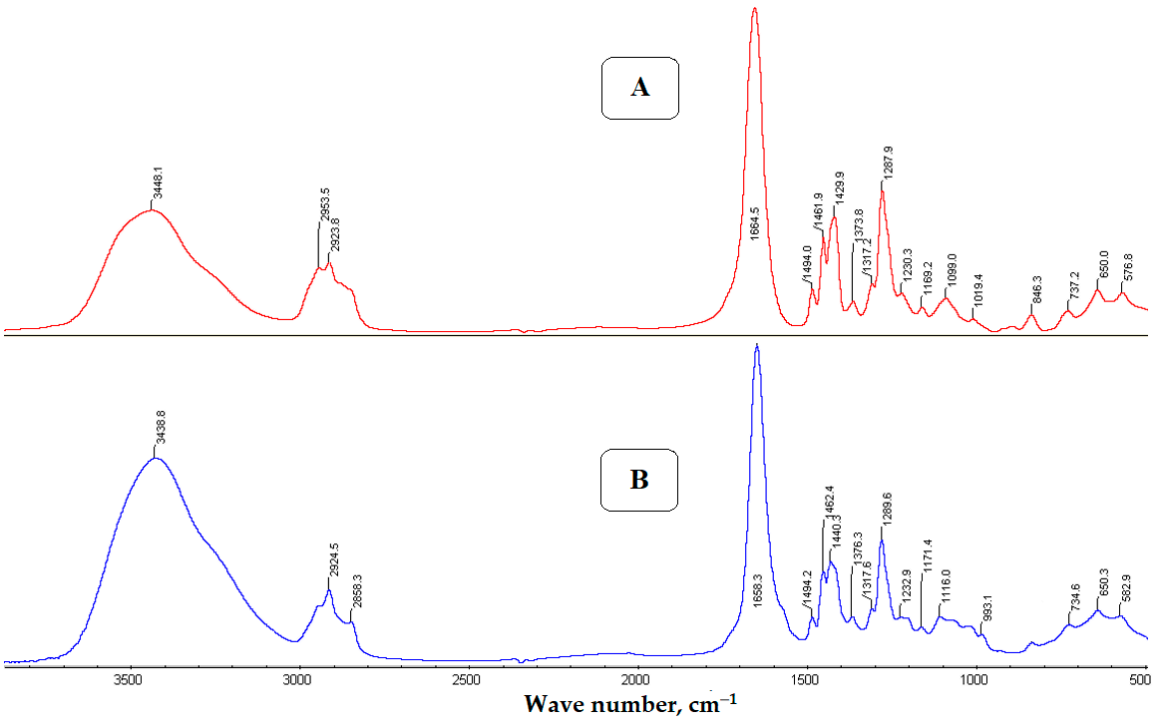
IR spectrum recorded in tablets of potassium bromide: (**A**) amphiphilic copolymer of *N*-vinyl-2-pyrrolidone and allyl glycyl ether; (**B**) copolymer of *N*-vinyl-2-pyrrolidone and allyl glycyl ether containing immobilized doxorubicin.

In the IR spectra of the synthesized amphiphilic copolymer of *N*-vinyl-2-pyrrolidone and allyl glycyl ether (Figure 2A), signals characteristic of poly(*N*-vinyl-2-pyrrolidone) are clearly manifested, and there are also signals at 1049.5 and 1089.7 cm^−1^ caused by asymmetric C-O-C stretching vibrations of the ether fragment, as well as bands at 845.4 cm^−1^ and 881.4 cm^−1^ corresponding to symmetric skeletal vibrations of the epoxy ring. The presence of these signals is associated with the presence of allyl glycidyl ether residues in the polymer chain [57].

**Figure 3:**
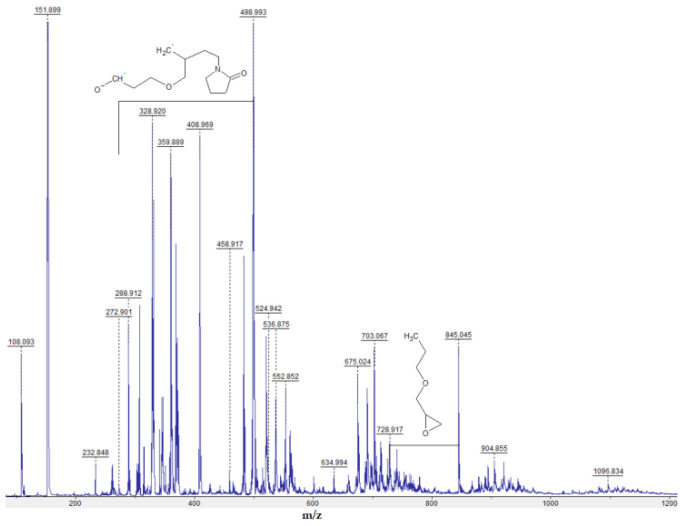
MALDI spectrum of an amphiphilic copolymer of *N*-vinyl-2-pyrrolidone and allyl glycyl ether obtained under anion detection conditions.

MALDI spectra with the detection of positively and negatively charged ions were also consistent with the structure of the resulting amphiphilic copolymer. The MALDI spectra with registration of negatively charged particles predominantly detect the degradation products of the amphiphilic copolymer (Figure 3).

**Figure 4:**
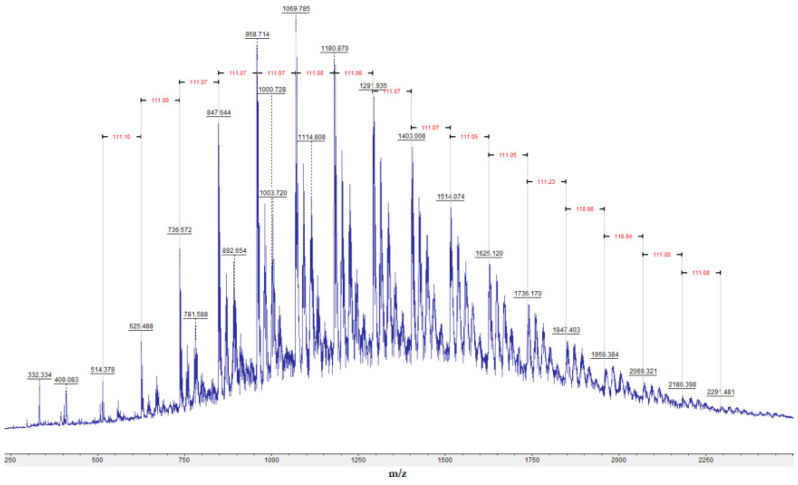
MALDI spectrum of the amphiphilic copolymer of *N*-vinyl-2-pyrrolidone and allyl glycyl ether acquired under cation registration conditions.

The MALDI spectra obtained under the conditions of registration of positively charged ions (Figure 4) are characterized by an m/z difference between the most intense signals close to 111.14, which corresponds to the compound repeating unit of *N*-vinyl-2-pyrrolidone.

The MALDI spectrum of the amphiphilic copolymer of *N*-vinyl-2-pyrrolidone and allyl glycyl ether obtained at a ratio of VP/AGE = 3 (Figure 4) is in qualitative agreement with its number-average molecular weight determined by end-group analysis, which is 1850.

**Figure 5:**
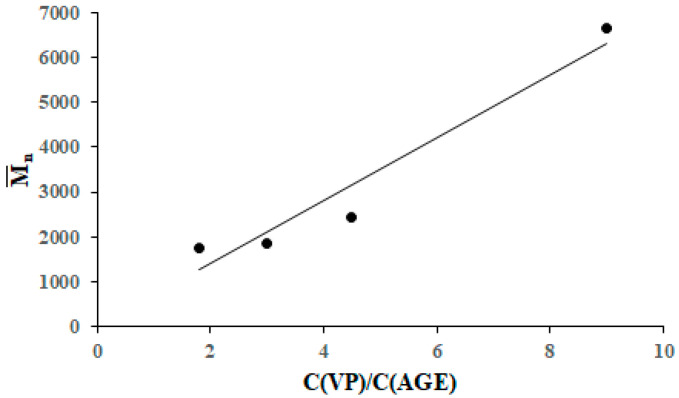
Dependence of the number-average molecular weight of the amphiphilic copolymers of *N*-vinyl-2-pyrrolidone and allyl glycidyl ether on the ratio of monomers in the reaction system.

The number-average degree of polymerization of amphiphilic copolymers increases almost linearly with an increasing VP/AGE concentration ratio in the reaction system (Figure 5). The obtained dependence indicates a lower rate of chain growth with the participation of allyl glycidyl ether compared to the rate of addition of *N*-vinyl-2-pyrrolidone and a significant contribution of chain transfer to allyl glycidyl ether. In this case, relation (3) must be satisfied:

(3) $$\bar{M_{n}}∼\frac{k_{p}C\left(R\right)C\left(\mathrm{VP}\right)}{k_{\mathrm{tm}}C\left(R\right)C\left(\mathrm{AGE}\right)}∼\frac{k_{p}C\left(\mathrm{VP}\right)}{k_{\mathrm{tm}}C\left(\mathrm{AGE}\right)},$$

where C(VP) and C(AGE) are the initial concentrations of *N*-vinyl-2-pyrrolidone and allylglycidyl ether in the reaction mixture, respectively; and k_p_ and k_tm_ are the effective rate constants of chain propagation with addition of *N*-vinyl-2-pyrrolidone and chain transfer to allyl glycidyl ether, respectively.

Linear regression, $\bar{M_{n}}$ vs. (C(VP))/(C(AGE)), yields Equation (4), which is characterized by a Fisher parameter of 33.38 and a tabular F-test value of 18.51 (α = 0.05).

(4) $$\bar{M_{n}}=723.66\frac{C\left(\mathrm{VP}\right)}{C\left(\mathrm{AGE}\right)}-136.26,$$

**Scheme 2:**
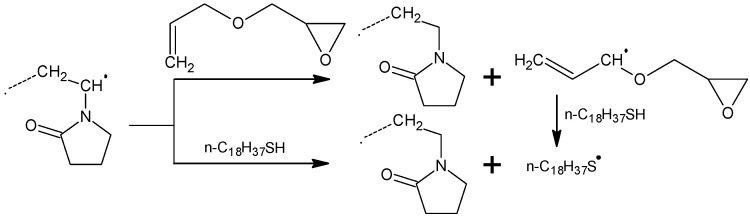
Chain transfer during the copolymerization of *N*-vinyl-2-pyrrolidone and allyl glycidyl ether in the presence of n-octadecylmercaptan.

Taking into account the high activity of allyl monomers in chain transfer and the passivity of allyl radicals in reinitiation [58], the mechanism of molecular weight regulation during the copolymerization of *N*-vinyl-2-pyrrolidone and allyl glycidyl ether in the presence of n-octadecylmercaptan can be represented by Scheme 2. Although the resulting the allyl radical is not sufficiently active in reinitiation, it can interact with n-octadecylmercaptan to form an octadecylthio radical active in reinitiation. The decisive role of chain transfer to allyl glycidyl ether is evidenced by the insignificant value of the free term of linear regression (4), which is consistent with the theoretical Equation (3).

**Figure 6:**
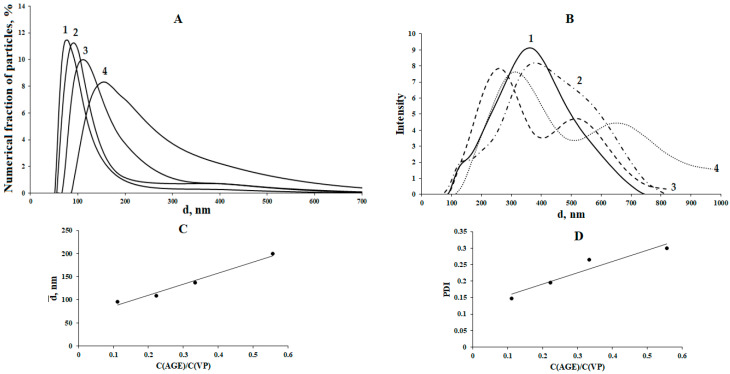
Plots of size vs. chain composition. (**A**) Number distributions of the aggregate diameters for different VP/AGE molar ratios: (1) −9; (2) −4.5; (3) −3; (4) −1.8. (**B**) Intensity distributions of the aggregate diameters. (**C**) Number-average particle diameter as a function of VP/AGE ratio. (**D**) PDI as a function of VP/AGE ratio.

Amphiphilic copolymers obtained at various VP/AGE ratios in the reaction mixture, when dissolved in water, are capable of forming nanosized aggregates as a result of self-assembly (Figure 6).

The number-average diameter of the aggregates increases rapidly with increasing proportion of AGE in the co-monomer mixture (Figure 6B) according to the linear regression Equation (5).

(5) $$\bar{d}=239.86\frac{C\left(\mathrm{AGE}\right)}{C\left(\mathrm{VP}\right)}+61.91,$$

Linear regression (5) has a Fisher test of 87.48 with a tabular F-test value of 18.51 (α = 0.05).

A decrease in the VP/AGE concentration ratio (Figure 5) leads to a decrease in the molecular weight of the hydrophilic block and, probably, its hydrophilicity. Obviously, a decrease in the molecular weight of the hydrophilic fragment leads to a decrease in the efficiency of the mechanism of steric stabilization of aggregates. It can also be assumed that an increase in the proportion of AGE residues in the chain leads to an increase in interchain hydrophobic interactions. Therefore, with an increase in the mole fraction of AGE in the initial reaction mixture, both the number-average particle diameter (Figure 6C) and PDI increased (Figure 6D). The presence of interchain hydrophobic interactions is supported by the transition to a pronounced bimodal intensity distribution of the aggregate diameters, along with the growth of the allyl glycidyl ether ratio in the copolymer (Figure 6B). 

**Figure 7:**
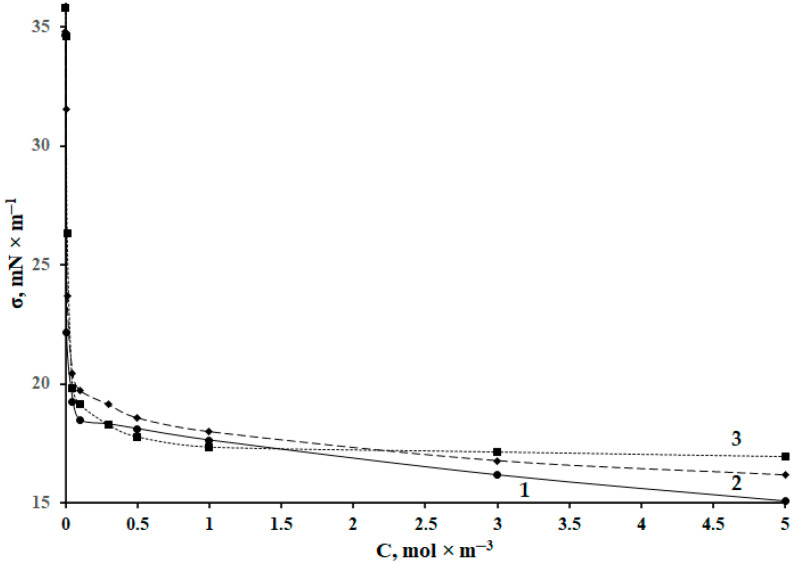
Surface tension isotherms at the water/toluene interface for amphiphilic copolymers of *N*-vinyl-2-pyrrolidone and allyl glycidyl ether obtained at various VP/AGE molar ratios in the reaction mixture: (1) −9; (2) −4.5; (3) −3.

Surface tension isotherms at the water/toluene interface for copolymers obtained at different ratios of VP and AGE are shown in Figure 7.

With a change in the concentration of amphiphilic copolymers of *N*-vinyl-2-pyrrolidone and allyl glycidyl ether in a narrow range, a rapid decrease in surface tension to 15–16 mN·m^−1^ is observed due to adsorption of the copolymer on the interface (Figure 7). The critical aggregation concentration (CAC) increases with decreasing hydrophilicity and hydrophilic block length (Table 1).

A decrease in the VP/AGE ratio in the reaction system leads to the suppression of core formation in aggregates due to disruption in the packing regularity of hydrophobic fragments. This can be attributed to hydrophobic interactions of terminal n-octadecyl groups and AGE residues. An increase in the proportion of *N*-vinyl-2-pyrrolidone in the reaction mixture leads not only to an increase in molecular weight but also an increase in the proportion of extended chain conformations in the formed copolymers, which manifests itself as an increase in S_0_ (Table 1).

**Scheme 3:**
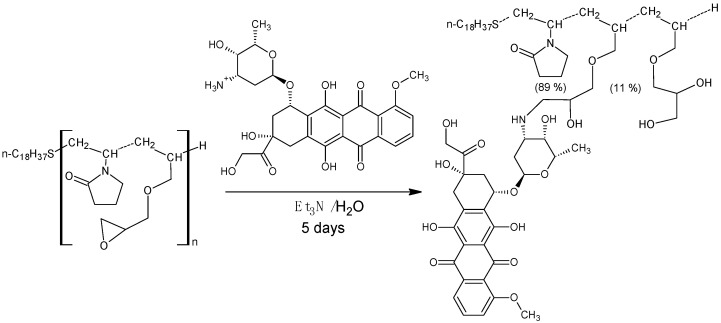
Covalent immobilization of doxorubicin on the amphiphilic copolymer of *N*-vinyl-2-pyrrolidone and allyl glycidyl ether.

The resulting amphiphilic copolymers of *N*-vinyl-2-pyrrolidone and allyl glycidyl ether are capable of simultaneous immobilization of doxorubicin and paclitaxel. Paclitaxel is incorporated into the core of the aggregates via hydrophobic interactions, whereas the immobilization of doxorubicin is covalent (Scheme 3).

**Figure 8:**
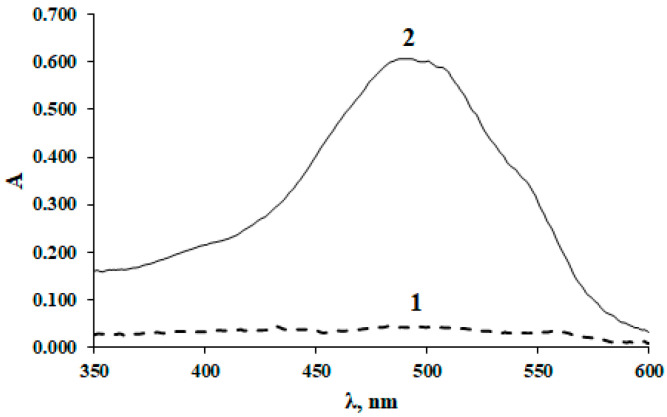
UV-vis spectrum of aqueous solutions as follows: (1) an amphiphilic copolymer of *N*-vinyl-2-pyrrolidone and allyl glycidyl ether; (2) an amphiphilic copolymer of *N*-vinyl-2-pyrrolidone and allyl glycidyl ether containing immobilized doxorubicin.

The UV-vis spectrum of an aqueous solution of an amphiphilic copolymer of *N*-vinyl-2-pyrrolidone and allyl glycidyl ether containing immobilized doxorubicin at a concentration of 1.221 g/L has an optical density of 0.587 at a wavelength of 480 nm (Figure 8). Given the molar absorption coefficient of doxorubicin of 13,500 L·mol^−1^·cm^−1^ [59] at a wavelength of 480 nm and the molar fraction of allyl glycidyl ether units in the amphiphilic copolymer of ca. 11% according to reverse acid–base titration, the reaction yield was 16%. This value is close to the content of epoxy groups in the copolymer of *N*-vinyl-2-pyrrolidone and allyl glycidyl ether obtained at the same VP/AGE molar ratio but in the absence of *n*-octadecylmercaptan [60].

**Figure 9:**
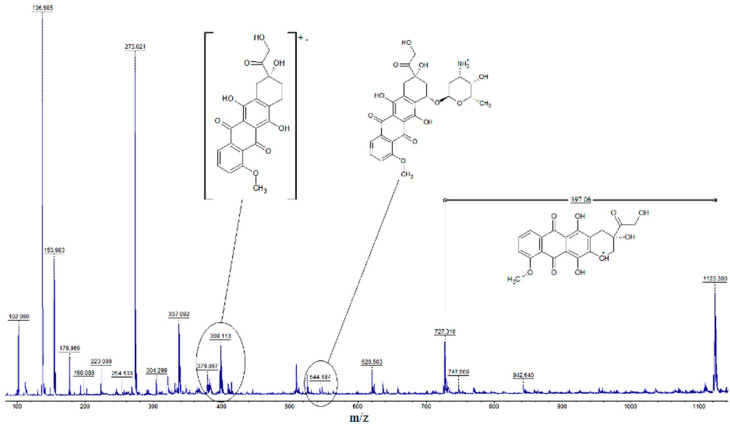
MALDI spectrum of an amphiphilic copolymer of *N*-vinyl-2-pyrrolidone and allyl glycidyl ether containing immobilized doxorubicin.

**Scheme 4:**
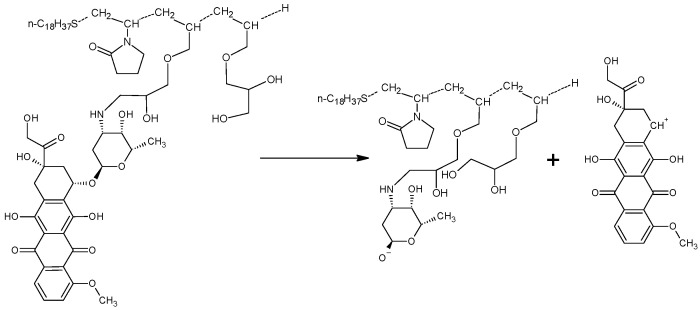
Fragmentation of an amphiphilic copolymer of *N*-vinyl-2-pyrrolidone and allyl glycidyl ether containing immobilized doxorubicin under laser desorption conditions.

The covalent immobilization of doxorubicin on the amphiphilic copolymer of *N*-vinyl-2-pyrrolidone and allyl glycidyl ether was confirmed by the presence of 1123.39 and 727.31 peaks in the MALDI spectrum, with an *m/z* difference of 397.06 (Figure 9), which corresponds to the cleavage of the anthracycline fragment (Scheme 4). The IR spectroscopy data indicate the appearance of new absorption bands observed in the wavenumber range of 1000–1100 cm^−1^ (Figure 2B). The absorption bands appear to correspond to the stretch vibrations of the C-O-bonds of the glycol fragments forming as a result of the hydrolysis of the epoxy groups that occurs in parallel with the covalent immobilization of doxorubicin.

**Figure 10:**
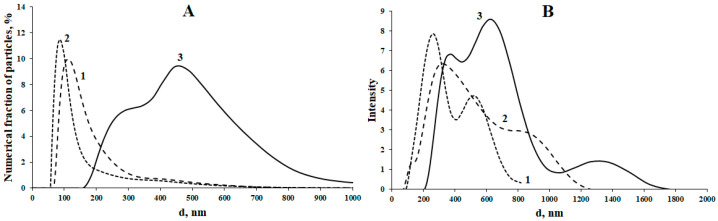
Number (**A**) and intensity (**B**) distributions of aggregate sizes: (1) initial amphiphilic copolymer (VP/AGE = 3); (2) after immobilization of doxorubicin; (3) after subsequent incorporation of paclitaxel.

Covalent immobilization of doxorubicin on the amphiphilic copolymer of *N*-vinyl-2-pyrrolidone and allyl glycidyl ether (VP/AGE = 3) leads to a slight decrease in the aggregate number-average diameter from 136 nm to 106 nm (Figure 10). Presumably, a slight decrease in the number-average diameter after immobilization of doxorubicin can be explained by partial dehydration of the particle shell, as lower affinity to water is expected for immobilized doxorubicin compared to *N*-vinyl-2-pyrrolidone residues. The intensity-average particle diameters increased from 293 nm to 358 nm, whereas the PDI was increased from 0.265 to 0.330. The subsequent incorporation of paclitaxel into the nanosized aggregates with immobilized doxorubicin leads to an increase in the number average particle diameter up to 410 nm (the intensity-average particle diameter was 518 nm), as well as the formation of a fraction of particles of more than 1 µm in diameter, which corresponds to free paclitaxel not incorporated into the aggregates of the amphiphilic copolymer (the latter can be removed by filtration). Furthermore, there are two fractions of particles with a diameter below 1 µm. The fraction of the 200–400 nm particles corresponds to the initial aggregates of the *N*-vinyl-2-pyrrolidone and allyl glycidyl ether copolymer chains, whereas the 400–800 nm fraction corresponds to secondary aggregation of the initial nanoparticles. Although the particle size distribution is broad, this did not cause any significant hindrances because the system remained stable, and no precipitation of particles from the dispersion was observed. Given the molar absorption coefficient of paclitaxel of 29,800 L·mol^−1^·cm^−1^ at a wavelength of 227 nm [61], paclitaxel loading was about 65%, as determined by photometry.

Numerous studies show that due to the high hydrophobicity and negligible solubility of paclitaxel in water, the process of its release from the hydrophobic cores of nanosized aggregates takes days [62,63,64]. We recently showed that the uptake by U87 and CRL 2429 cells of aggregates of a structurally similar amphiphilic *N*-vinyl-2-pyrrolidone homopolymer loaded with a hydrophobic DiI dye is very efficient and reaches saturation within few hours [20,21]. Given the high uptake rate of amphiphilic poly(*N*-vinyl-2-pyrrolidone) aggregates by cells, paclitaxel appears to be involved in metabolic processes prior to its passive release from nanoscale aggregates via diffusion. 

In this article, doxorubicin and paclitaxel were used as model drugs to demonstrate the possibility of dual loading into nanoaggregates of an amphiphilic copolymer of *N*-vinyl-2-pyrrolidone and allyl glycidyl ether. The demonstrated approach is rather general; the synthesized polymeric nanocarrier is expected to be applicable for dual loading and co-delivery of other combinations of pharmacologically active substances, provided one component contains an amino group and the other is sufficiently hydrophobic to be included into the aggregate core.

## 4. Conclusions

Novel nanocarriers self-assembled from amphiphilic copolymers of *N*-vinyl-2-pyrrolidone and allyl glycidyl ether containing a terminal n-octadecylthio group were synthesized and characterized. Synthesis of the amphiphilic copolymers was carried out by a radical copolymerization reaction with AIBN as an initiator and in the presence of n-octadecylmercaptan as a chain transfer agent. It has been shown that the molecular weight of the amphiphilic copolymers is determined by chain transfer to allyl glycidyl ether. Reinitiation occurs only with n-octadecylthio radicals formed as a result of the interaction of n-octadecylmercaptan with passive allyl radicals and primary degradation products of AIBN. With an increase in the proportion of allyl glycidyl ether, the size of aggregates formed by amphiphilic copolymers increases. The obtained nanocarriers are shown to be capable of dual loading of common antitumor drugs doxorubicin and paclitaxel. Covalent immobilization of doxorubicin occurs at the epoxy group of allyl glycidyl ether residues in the amphiphilic copolymer and is accompanied by a slight decrease in aggregate size. On the contrary, the loading of paclitaxel in the hydrophobic core of the aggregates leads to a significant increase in the aggregate size and broadening of the size distribution. The described loading strategy can be straightforwardly extended to dual loading of other amine-containing and hydrophobic drugs. The synthesized amphiphilic copolymer of *N*-vinyl-2-pyrrolidone and allyl glycidyl ether could be of interest for the co-loading and co-delivery of a wide range of pharmacological preparations and can lead to new, more efficient combination therapies with reduced cardiotoxicity. 

## Figures and Tables

**Table 1:** Properties of surfactants based on amphiphilic copolymers of *N*-vinyl-2-pyrrolidone and allyl glycidyl ether containing a terminal *n*-octadecyl group.

VP/AGE	${\bar{M}}_{n}$	Г_max_ × 10^−7^ mol·m^−2^	S_0_ × 10^18^, m^2^	δ × 10^9^, m	CAC, mol·m^−3^
9	6700	7.1	2.34	4.32	0.0057
4.5	2430	7.99	2.08	1.74	0.0157
3	1850	16.5	1.01	2.7	0.0285

## Data Availability

Data available upon request to the corresponding author.
